# The molecular structure of β-alanine is resistant to sterilising doses of gamma radiation

**DOI:** 10.1371/journal.pone.0210713

**Published:** 2019-01-15

**Authors:** Lívia de Souza Gonçalves, Mariana Franchi, Monica B. Mathor, Ademar B. Lugao, Victor H. Carvalho, Marisa H. G. Medeiros, Guilherme Giannini Artioli, Gustavo H. C. Varca

**Affiliations:** 1 Applied Physiology & Nutrition Research Group, Rheumatology Division, Faculdade de Medicina FMUSP, Universidade de São Paulo, São Paulo, SP, Brasil; 2 Instituto de Pesquisas Energéticas e Nucleares, IPEN-CNEN/SP, São Paulo, SP, Brasil; 3 Departamento de Bioquímica, Instituto de Química, Universidade de São Paulo, São Paulo, SP, Brasil; North Shore Long Island Jewish Health System, UNITED STATES

## Abstract

β-alanine is the rate-limiting point for the endogenous synthesis of carnosine in skeletal muscle. Carnosine has a wide range of implications for health, normal function and exercise performance. Whilst the physiological relevance of carnosine to different tissues remains enigmatic, β-alanine administration is a useful strategy to investigate the physiological roles of carnosine in humans. Intravenous administration of β-alanine is an interesting approach to study carnosine metabolism. However, sterilisation is mandatory due to the nature of the administration route. We evaluated whether sterilising doses of gamma radiation damages the molecular structure and leads to the loss of functional characteristics of β-alanine. Pure β-alanine was sterilised by gamma radiation in sealed glass vials using a ^60^Co multipurpose irradiator at a dose rate of 8.5 kGy.hour^-1^ totalising 10, 20, 25 30 and 40 kGy. The molecular integrity was assessed by X-ray Diffraction and changes in content were determined by High Performance Liquid Chromatography (UV-HPLC) and Triple Quadrupole Mass Spectrometer (HPLC/MS-MS). Sterility assurance was evaluated by inoculation assay. To examine whether functional properties were preserved, β-alanine was infused in one participant, who rated the level of paraesthesia on the skin using a 0–3 scale. Urinary β-alanine was quantified before and 24-h following β-alanine infusion using HPLC-ESI+-MS/MS. Irradiation resulted in no change in the crystal structure of β-alanine, no degradation, and no new peaks were identified in the dose range assayed. The inoculation assay showed the absence of viable microorganisms in all β-alanine samples, including those that did not undergo irradiation. Intravenous infusion of β-alanine resulted in paraesthesia and it detected in the urine as per normal. We conclude that gamma radiation is a suitable technique for the sterilisation of β-alanine. It does not lead to degradation, damage to the β-alanine structure, content or loss of function within the evaluated irradiation conditions.

## Introduction

β-alanine is the β-amino acid most commonly found in bioactive peptides [1], such as carnosine and its methylated analogues anserine and balenine Dunnett and Harris [2]. Carnosine and its methylated analogues are abundantly found in skeletal muscle of many vertebrates; in human omnivores, typical values range from ~10 to ~40 mmol^.^kg^-1^ of dry muscle [3–5]. Carnosine is also highly expressed in other tissues, such as cardiac muscle [6] and in some regions of the brain [7, 8]. It has been suggested that carnosine plays several different key physiological roles, such as pH regulation [9, 10], scavenging of reactive species, protection against glycation [11, 12] and regulation of calcium sensitivity and calcium transient in the contractile apparatus of the skeletal [13, 14] and cardiac muscles [15]. Therefore, carnosine has been considered a compound of relevance to the biomedical sciences, with potential applications to health and performance [16–18].

The intramuscular synthesis of carnosine is limited by the low availability of β-alanine [19]. Moreover, skeletal muscle does not seem to express the carnosine transporters PEPT1 and PEPT2 [20], meaning that the uptake of carnosine in its intact form into skeletal muscle is substantially low or non-existent. In addition, the concentrations of carnosine in blood are normally below the micromolar limits of detection of chromatographic techniques [19], which is a consequence of the high activity of the carnosinase enzyme present in plasma [21]. Thus, increasing the availability of β-alanine to tissues via supplementation appears to be an effective way to increase tissue levels of carnosine. In fact, β-alanine has been consistently shown to increase muscle carnosine content in young [5, 19] and old individuals [22]; this is typically accompanied by improved muscle function and exercise tolerance [22, 23]. However, it is still uncertain whether β-alanine administration can increase carnosine content in other tissues [24]. Tissue carnosine content is dependent on total of β-alanine consumed [5, 25]. When large amounts (>1600 mg) of β-alanine are ingested, plasma β-alanine concentrations sharply increase and paraesthesia develops (i.e., individuals report itch and tingling sensations on the skin) [19]. Such effect is triggered when β-alanine binds to MrgprD, a membrane receptor that is abundantly found in sensory neurons that exclusively innervate the skin [26]. Approximately 1.5 to 3% of the β-alanine ingested orally is excreted in the urine [19, 27] with the remaining being stored in different body compartments or oxidised [28]. Despite the growing interest on the functional and therapeutic effects of carnosine in skeletal muscle and in other tissues, several questions about fundamental physiologic processes involving β-alanine and carnosine metabolism remain unanswered.

In the field of physiology, intravenous infusion has long been used as a powerful tool so study mechanisms underlying the uptake, metabolism and degradation of a variety of substances [29–32]. In that regard, intravenous infusion of β-alanine could be a controlled manner to examine rates of β-alanine incorporation into tissues, and to investigate strategies to optimise uptake, for example. However, ensuring sterility is mandatory for the intravenous administration of any compound. Although several methods of sterilisation are available [33], it is crucial to choose a method that is completely efficient and compatible with the intended application, without compromising the molecular structure or degrading the compound.

Gamma radiation is a highly efficient method of sterilisation whose effectiveness lies on its high penetration in matter and high capacity to inactivate microorganisms. Advantages of gamma radiation are 1) sterilisation occurs without rising product temperature, 2) it is carried out in a completely dry process, and 3) allows the material to be sterilised in its final packaging, preventing post-sterilisation recontamination [34–36]. However, high doses of gamma radiation may modify physical, chemical and biological properties of products [34]. Amino acids appear to be particularly sensitive to ionising radiation, since previous studies have shown fragmentation and oxidation [37–39]. Gottschall & Tolbert [40] showed that α-alanine, a β-alanine isomer, underwent significant degradation following exposure to gamma radiation in an absorbed dose-dependent manner, which might have occurred through deamination and decarboxylation.

The isomers α/β-alanine present a few key differences in their structural arrangements that may result in different behaviour under exposure to radiation. Abirami et al. [41] suggested that the intramolecular hydrogen bonding patterns are different between α- and β-alanine. In addition, the presence of a methylene group (-CH2-) in the β position of β-alanine appears to offer enhanced flexibility when compared to α-alanine. Although these differences suggest that β-alanine may exhibit low sensitivity and could be more resistant to degradation, the β-alanine behaviour under high-energy irradiation, and therefore its suitability for being sterilised with this method, remains unexplored. In the present study, we examined the use of gamma radiation for β-alanine sterilisation focusing on the subsequent use in human for intravenous infusion; the molecular integrity and functionality of β-alanine were assessed before and after increasing sterilising doses of gamma radiation.

## Materials and methods

### Sample preparation

Commercially available β-alanine in powder form (99%, molecular weight 89.09 g.Mol^-1^ obtained from Sigma-Aldrich, USA) were transferred to glass vials (1 g per vial), which were hermetically sealed and sterilised by gamma radiation. Control samples were also transferred to identical vials and submitted to the same process, except the irradiation, to serve as non-irradiated negative controls.

### Gamma radiation

β-alanine was irradiated at room temperature using a ^60^Co multipurpose irradiator (IPEN-CNEN/SP, Brazil) at a dose rate of 8.5 kGy.hour^-1^ to reach the standardised dose for sterilisation of 25 kGy and total absorbed doses of 10, 20, 30 and 40 kGy. Dosimetry was performed using Harwell Type 4034 dosimeters. Three independent vials containing 1g of β-alanine were submitted to each of the radiation doses.

### β-alanine quantification

#### HPLC

β-alanine samples and controls were quantified by HPLC (Hitachi, Hitachi Ltd., Tokyo, Japan) according to the method described by Mora et al. [42]. Ten microliters of each β-alanine sample (50 mM) were injected using an autosampler equipped with an aspiration sipper tube. The chromatographic separation was developed using the Atlantis HILIC silica column (4.6 x 150 mm, 3 μm) from Waters (Milford, MA) at room temperature. Mobile phases consisted of solvent A, containing 0.65 mM ammonium acetate, pH 5.5, in water/acetonitrile (25:75), and solvent B, containing 4.55 mM ammonium acetate, pH 5.5, in water/acetonitrile (70:30). The solvents were filtered through a 0.22 μm membrane filter and degassed prior to the analytical run. The separation condition was a linear gradient from 0 to 100% of solvent B in 13 min at a flow rate of 1.4 ml.min^-1^. Separation was monitored using a UV detector at a wavelength of 214 nm for β-alanine detection. Peak areas were used to quantify compound concentration by interpolation in the corresponding calibration curve. All samples and standards were quantified in duplicates.

#### HPLC/MS-MS

β-alanine samples and controls were analysed by HPLC (Agilent series 1100, Agilent, USA) using Ascentis express column C18 (75 mm x 2.1mm, 2.7 μm) from Sigma-Aldrich (USA) at room temperature. Mobile phases consisted of solvent A, containing water and 0.1% of formic acid, and solvent B, containing acetonitrile and 0.1% of formic acid, isocratic mode: 50% solvent A and 50% solvent B, and flow rate of 0.4 ml.min^-1^. The injected volume was 5 μl. Mass spectrometry was performed on a Triple Quadrupole device (MS/MS) (API 2000, Sciex, Concord, Canada) with electrospray source operated in negative ion mode. The electrospray source parameters were as follows: nebuliser gas = 60 psi, heater gas = 50 psi, heater temperature = 350 ^º^C, and electrospray voltage = + 4200V. The SRM mode was used to detect the β-alanine in the triple quadrupole stage.

Samples were prepared by diluting a 4 mg.ml^-1^ stock solution in an 80% water and 20% acetonitrile solution (v/v). For working solution, 12.5 μl of each solution was diluted in 487.5 μl of the dilution solution.

Derivatisation of β-alanine samples was performed using 50 μl phenyl isothiocyanate reagent 50 μl (0.1 M) and 50 μl triethylamine (1.0 M) followed by vortexing for 10 seconds and incubation at 40°C under stirring (350 rpm) for 20 minutes. Then, 200 μl of n-hexane (clean up) was added and vortexed again for 30 seconds. 200 μl of the aqueous phase was transferred to the vial containing 980 μl of dilution solution. The samples were then injected into the HPLC/MS-MS system. All samples were analysed in unicates.

### Crystallinity of β-alanine

X-ray Diffraction studies were conducted on a Multiflex difractometer (Rigaku, Japan) at a geometric speed of 1°/min, at 2θ scale ranging from 5 to 60 ^o^C in an λ = 1,54184 Å. The spectra of the β-alanine samples in unicates were treated with Search-Mach and in accordance to the PDF-ICDD database.

### Sterility testing

Sterility assurance was tested by an independent accredited company (Controlbio Technical Assistance Microbiological) using the direct inoculation assay as described in the US Pharmacopeia and in accordance to the ISO 11137 [43]. Each sample in unicate was inoculated on a culture medium and incubated for 14 days with daily verification.

### Assessment of functional characteristics of β-alanine in humans

In order to examine whether the functionality of β-alanine following exposure to gamma radiation remained preserved, we infused 0.8mg^.^kg^-1.^ml^-1^ of β-alanine for 180 minutes, totalising 11g of β-alanine, in a young, healthy men who volunteered for this experiment. Since all tested doses resulted in no change in β-alanine structure and content, we opted to use 25 kGy dose of gamma radiation to sterilise the β-alanine samples used for infusion since this a validated standard dose to sterilise medical products for intravenous infusion [44]. A midstream urine sample was collected before infusion and a post-infusion 24-h urine sample was collected to confirm that β-alanine would be excreted and detected in human urine [19, 27], therefore showing preserved metabolic routes. Urine samples and standards were quantified in duplicates and analysed by on-line HPLC-ESI^+^-MS/MS, as described by Carvalho et al. [45] using CAR-*d*_*4*_ as internal standard. β-alanine was not derivatized prior to analysis and the SRM transitions monitored were m/z 90→72 (quantification transitions), m/z 90→45 and m/z 90→30 (confirmation transitions). The quantification transition m/z 90→72 is unique to β-alanine and can be used to differentiate the α- from the β-isomer [46]. To further confirm that irradiated β-alanine would exert its normal physiological effects, the participant was asked to rate the intensity of the paraesthesia he was experiencing every 10 min during the initial 30 minutes of infusion and then every 30 min until the end of infusion period. The intensity of paraesthesia was rated using a 0–3 scale as described by Lingjaerde et al. [47]. The experiment was approved by the Ethics Committee of School of Medicine, University of Sao Paulo (project #1185971). After being fully explained about the risks and benefits involved with participation, the participant signed the informed consent term and was obtained after a thorough explanation of the risks and benefits involved with the participation in the study.

### Statistical analysis

Due to the nature of this investigation, no inferential statistical tests were necessary to test our hypotheses. Repeated-measures coefficient of variation (CV) was calculated by dividing SD by the group mean. Data is presented as mean ± standard deviation (SD).

## Results

High Performance Liquid Chromatography (HPLC) was used to determine β-alanine concentration in solutions prepared with pure β-alanine samples that were submitted to doses of radiation of up to 40 kGy, as well as with non-irradiated β-alanine. No measurable loss or degradation were observed in β-alanine samples at solid state exposed to gamma radiation of up to 40 kGy, as determined via HPLC quantification of 50 mM solutions (Table 1).

**Table 1:** Mean ± standard deviation (SD) of the three β-alanine samples sterilised at each gamma irradiation dose. Coefficients of variation (CV) were calculated within a given irradiation dose based on the three values. 50 mM solutions were prepared for all samples and the percentage values in brackets refers to the differences in relation to a 50 mM reference concentration.

Irradiation dose (kGy)	β -alanine content mM (%)	CV (%)
10kGy	50.1 ± 0.5 (100%)	1.1
20kGy	50.0 ± 0.2 (100%)	0.3
25kGy	49.7 ± 0.7 (99%)	1.4
30kGy	49.1 ± 1.6 (98%)	3.3
40kGy	49.2 ± 1.1 (98%)	2.2

Dose rate = of 8.5 kGy hour^-1^; CV = coefficient of variation

In addition to the HPLC measurements, we evaluated the structural integrity of β-alanine. Liquid Chromatography coupled to Mass Spectrometry (HPLC/MS-M) was used in the same samples to identify the presence of any non-β-alanine-related peaks, as well as to confirm HPLC findings.

**Fig 1:**
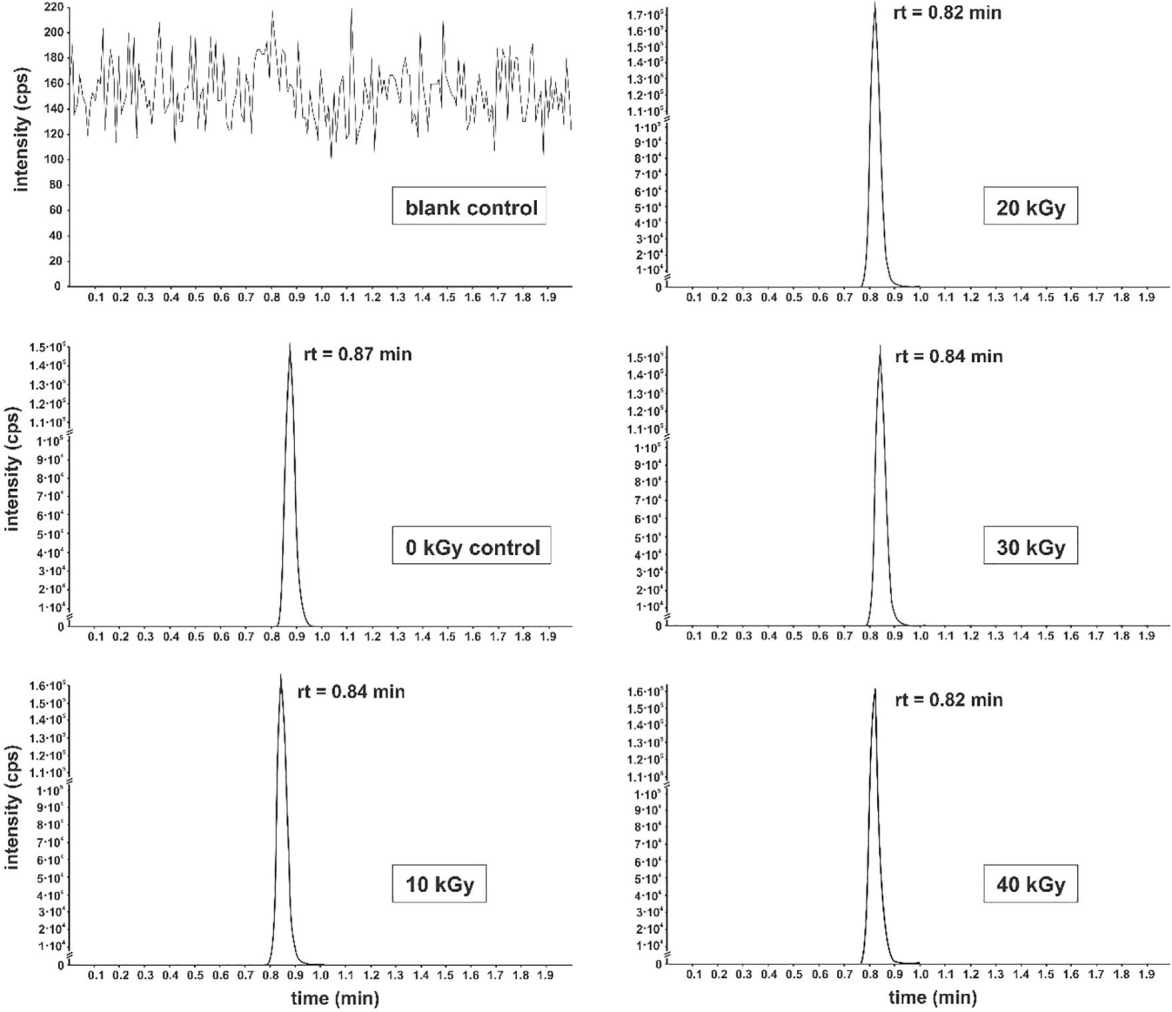
Representative chromatograms of selected reaction monitoring (SRM) of β-alanine after gamma irradiation at dose rate of 8.5 kGy hour^-1^ and absorbed doses ranging from 0 to 40 kGy. Chromatograms were obtained in a blank control, a non-irradiated 50 mM control, and 10–40 kGy irradiated 50 mM β-alanine samples.

The lack of degradation was further confirmed via HPLC-MS/MS which revealed no reductions in peak area, no unexpected peaks and no changes in chromatograms (Fig 1). The retention times registered for the samples were found to be very similar to the non-irradiated β-alanine thus highlighting amino acid integrity.

The examination of peak areas obtained by the HPLC-MS/MS confirmed that the signal registered for the irradiated samples corresponded to approximately 100% of the signal of the non-irradiated β-alanine control (Table 2).

**Table 2:** β-alanine concentration analysed in non-irradiated (control) and irradiated 50 mM samples. Analyse was performed using the chromatograms peak areas.

Dose	Peak area (counts)	β-alanine (% of the control)
0kGy (control)	4.09E+05	100.0
20kGy	4.06E+05	93.0
25kGy	4.60E+05	112.0
30kGy	4.19E+05	102.0
40kGy	4.43E+05	108.0

Dose rate = of 8.5 kGy hour^-1^

**Fig 2:**
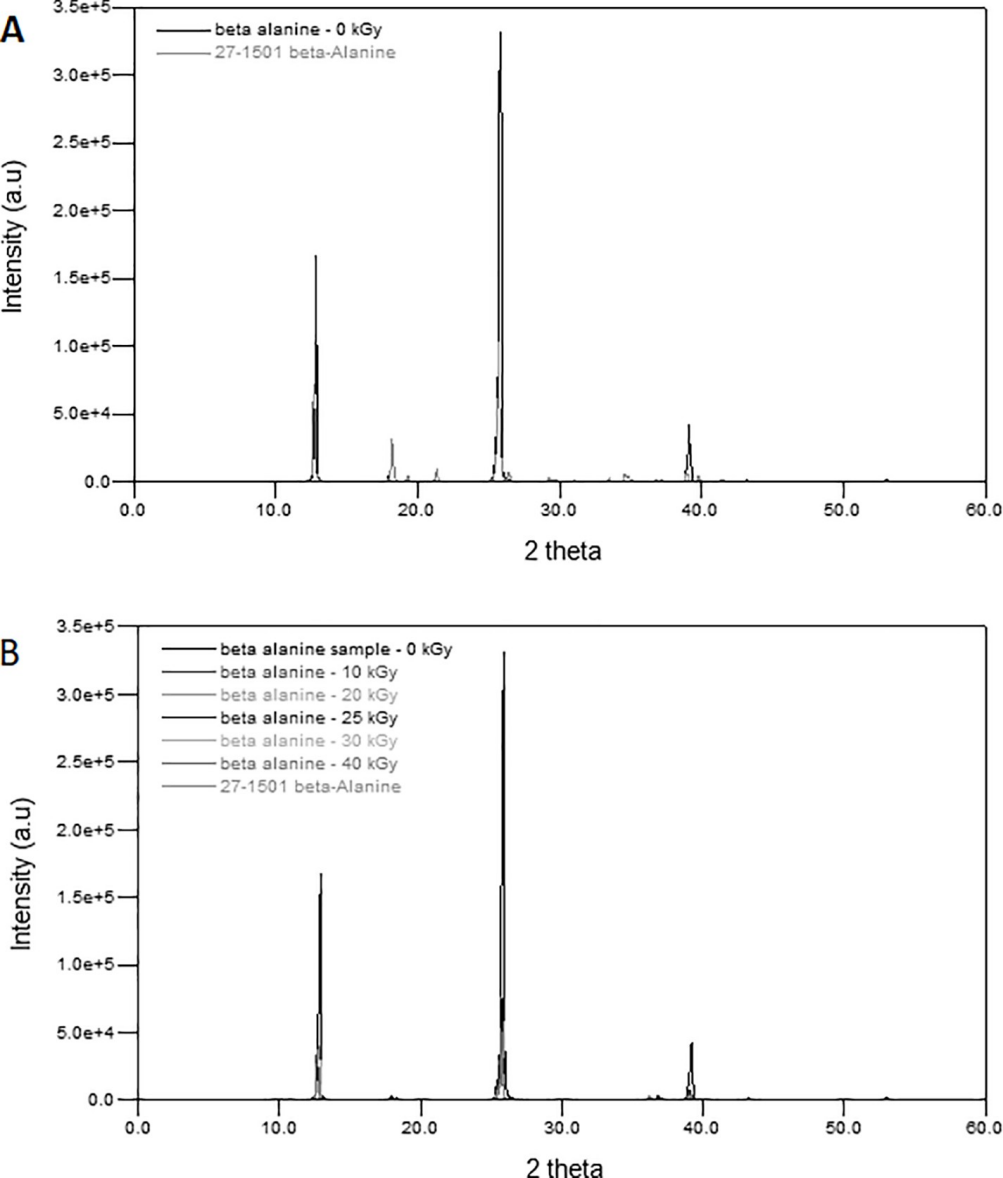
X-ray Diffractograms of β-alanine and β-alanine standard (27–1501—PDF-ICDD) before (a) and after gamma irradiation at dose rate of 8.5 kGy hour^-1^ and absorbed doses ranging from 10 to 40 kGy (b).

The non-irradiated an irradiated β-alanine samples were analysed with X-ray Diffraction (XRD) to access and track down changes in the crystalline structure of β-alanine. X-ray Diffraction analysis showed that the diffractogram of β-alanine matched the standard diffractogram of β-alanine (27–1501) of the PDF-ICDD database (Fig 2A), therefore confirming its purity.

Likewise, the irradiated samples presented similar diffractogram as obtained for β-alanine, further indicating that irradiation did not change the structural integrity of the β-alanine molecule within the dose range assayed. Also, irradiation did not lead to the formation of new peaks at irradiation doses of up to 40 kGy (Fig 2B), again reinforcing that gamma radiation resulted in no changes in the crystal structure of β-alanine.

To test for sterility, all irradiated and non-irradiated samples were submitted to accredited tests to detect the presence of microorganisms. The microbiological assays showed a negative bioburden (i.e., no colony-forming units) in all β-alanine samples, including the non-irradiated control and the irradiated samples at all doses assayed.

**Fig 3:**
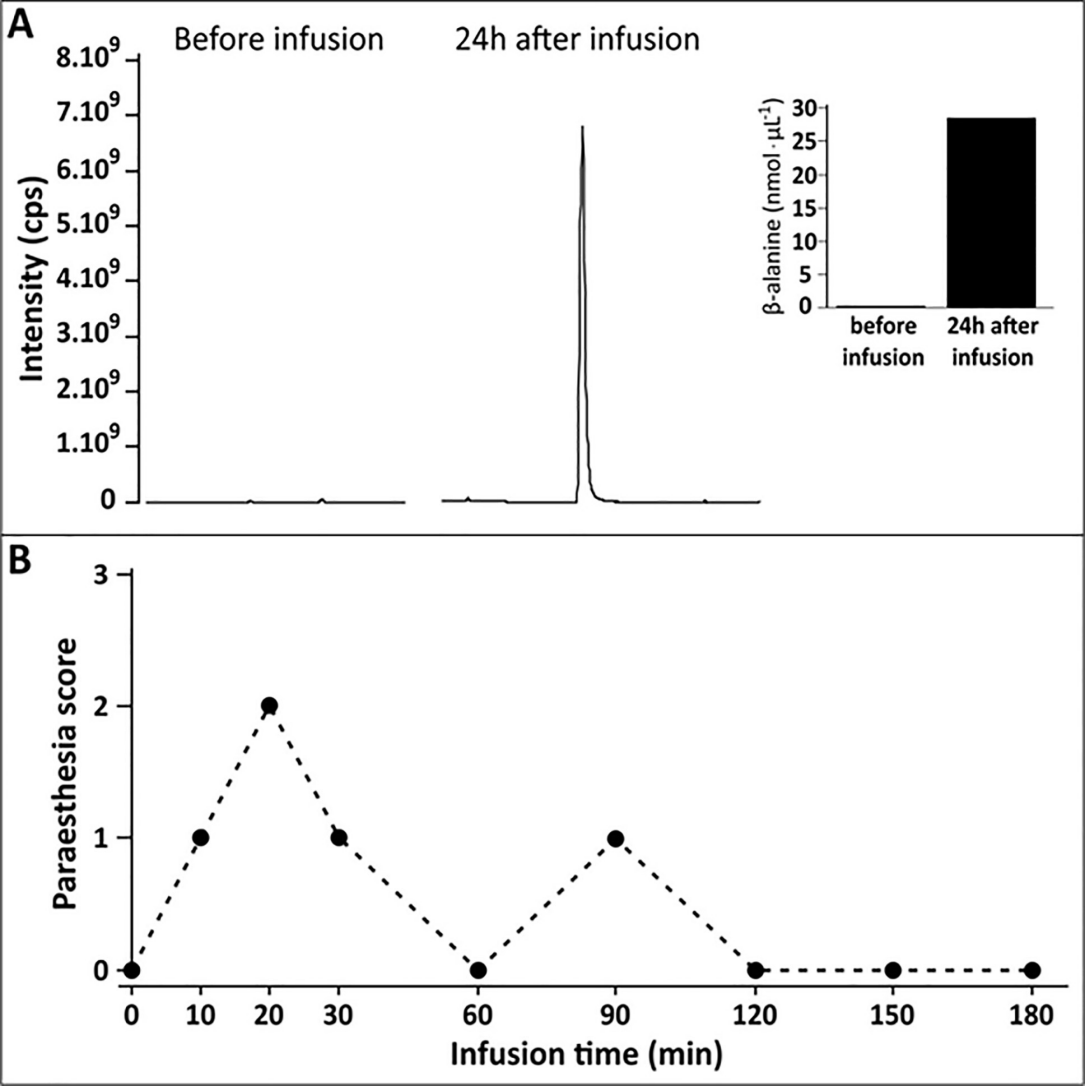
β-alanine peak intensities and concentrations obtained in a midstream urine sample before intravenous infusion of β-alanine and in a 24-h urine sample obtained after β-alanine infusion (Fig 2A). Symptoms of paraesthesia reported by the participant during intravenous infusion of irradiated β-alanine (Fig 2B).

β-alanine was not detected in the pre-infusion urine sample. However, high levels of β-alanine were detected and quantifies in the 24-h post-infusion urine sample (3.7g, or 34% of the total infused) (Fig 3A). Intravenous infusion of irradiated β-alanine in one participant elicited symptoms of paraesthesia at different time points throughout the infusion period (Fig 3B).

## Discussion

Gamma radiation is a powerful method of sterilisation. The inactivation of microorganisms when the product is irradiated in solid state occurs through the rupture of nucleic acids, changing their structure and function. However, the energy transferred by radioisotopes emitting gamma radiation results in a stream of electrons that can interact with atoms of the product under sterilisation. This process can induce excitation or displacement of electrons from their orbits, generating energy to interact with the electrons of other atoms. As a result, physical, chemical and biological properties of materials can be changed [34].

In the present study, we investigated whether β-alanine, an amino acid with several potential applications to human health and performance, may be submitted to sterilizing doses of ionising gamma radiation without undergoing degradation or changes in its chemical structure. Using radiation doses of up to 40 kGy, specially focusing in max/minimum dose, we herein demonstrated no changes in β-alanine content as well no changes in the spectrum profile, indicating no degradation and no chemical destructuring.

In contrast to our findings with β-alanine, Gottschall and Tolbert have shown that α-alanine undergoes degradation after exposure to high doses of ionising radiation [40, 48]. These researchers showed that after α-alanine irradiation by Cesium-37 gamma-source, ammonia was released in greatest yield upon dissolution andCO_2_ was the major gaseous product measured from the crystalline samples. It is plausible that the different results are related to the differences in the structural arrangement of these amino acids. Abirami et al. [41] have shown that the presence of a -CH2- group at the *β* position leads to physicochemical differences between β- and α-alanine that appears to offer greater structural flexibility to β-alanine.

Interestingly, β- and α-alanine seem to express different behaviours according to the type of energy to which they are exposed. Recently, Sousa et al.[49] showed that the β-alanine is more sensitive than α-alanine to high temperatures, which was attributed to the more acute intermolecular N–H•••O angle of β-alanine (149.2^o^); this may favour the rupture of the bonds, thus leading to earlier thermic decomposition when compared to α-alanine (angle is 164.2^o^). Although the molecular structure of α-alanine confers protection against the energy in high-temperature, the structural arrangement of β-alanine may protect it from the high energy imposed by radiation. In the present study, however, we could not determine the exact structural arrangement responsible for such increased protection.

In this investigation, we used two methods widely applied for the identification and quantification of compounds, namely HPLC and MS/MS. Both techniques were sufficiently robust to identify and quantify β-alanine. HPLC stands as a proper technique for separation and identification of compounds. However, HPLC requires a confirmatory technique when qualitative analysis (i.e. confirmation of chemical identity) is required. In this sense, MS/MS was applied to possibly identify the content and other possible degradation by-products of low or higher molecular weight. The lack of changes in both quantities and chromatograms qualities gives us a strong level of confidence to affirm that radiation doses of up to 40 kGy does not result in β-alanine degradation.

With regard to possible changes incurred by means of crystalline structure, the diffractograms revealed that the crystalline regions were preserved after irradiation, with no formation of new peaks. This indicates that there were no changes in the crystalline patterns traceable by XRD following gamma irradiation of up to 40 kGy. Therefore, the irradiation doses tested do not result in significant physical-chemical changes in β-alanine, which provides further experimental evidence to support gamma irradiation as a highly suitable method for β-alanine sterilisation.

It is known that the effectiveness of gamma radiation for sterilisation purposes relies on the amount of energy transferred and on the initial levels of contamination of the product. For parenteral solutions and instruments that are in direct contact with blood, the value of sterility assurance level (SAL) 10^−6^ is recommended (i.e., less than one out of million contaminants will survive on the product after sterilisation). According to ISO 11137 [43] a typical or standard sterilization dose is placed within 15 and 25 kGy with most products sterilised by radiation nowadays being validated for such doses. However, there is evidence in literature that irradiation doses of 25 kGy may change the chemical structure of certain compounds [50], which we herein demonstrated not to hold true in the case of β-alanine, even at doses up to 40 kGy.

The microbiological tests confirmed negative bioburden for non-irradiated β-alanine and irradiated β-alanine at all doses assayed. In specific terms, all samples were found to be sterile, including those that did not undergo irradiation. It is relevant to highlight that radiation is the only currently available technique that allows an estimation of the bioburden reduction of 10^-1^ at each 2.5 kGy. Based on such premise, at a zero bioburden or negative initial contamination level, it seems likely that a 15 kGy dose would already be enough to achieve the 10^−6^ SAL, therefore meeting the criteria established by ISO 11137 [44]. However, depending upon the contamination levels observed at each batch, higher doses may be required to reach a sterilization level within the 10^−6^ SAL. Nonetheless, β-alanine molecule was resistant to the sterilizing doses recommended by ISO 11137 and showed no degradation or transformation even at higher irradiation doses up to 40 kGy, indicating the suitability of gamma radiation even with high levels of initial contamination.

Finally, intravenous infusion of the irradiated β-alanine in one individual indicated that the its functional characteristics were not affected by exposure to gamma radiation. The symptoms of paraesthesia reported by the participant confirmed that the irradiated β-alanine preserved its ability to bind to the sensitive MrgprD receptors in a specific subset of sensory neurons at the human skin. These receptors are found in the dorsal root ganglia, responsible for innervation of the skin, making an important role in mediation of sensory perception signals, such us itch, one of the known manifestations of paraesthesia. Furthermore, the fact that β-alanine was quantified within the expected amounts in the urine, thus indicating that it is following its normal metabolic routes. Therefore, in addition to exhibiting preserved structural properties, β-alanine sterilised with 25 kGy also appears to preserve its functional properties in the human body.

## Conclusion

The results herein presented indicate that radiation for the sterilisation of β-alanine for intravenous administration may be successfully applied in accordance to the recommended standards with no expected relevant loss, change in the compound or loss of its functional characteristics. If required, higher gamma radiation doses up to 40 kGy may also be used without loss or degradation of the amino acid.
